# Formulation of a Ceramic Ink for 3D Inkjet Printing

**DOI:** 10.3390/mi12091136

**Published:** 2021-09-21

**Authors:** Dennis Graf, Judith Jung, Thomas Hanemann

**Affiliations:** 1 Laboratory for Materials Processing, University of Freiburg, 79110 Freiburg, Germany; thomas.hanemann@kit.edu; 2 Institute for Applied Materials, Karlsruhe Institute of Technology, 76344 Eggenstein-Leopoldshafen, Germany; judith.jung@kit.edu

**Keywords:** additive manufacturing, material jetting, polymer-ceramic composites, ceramic inks

## Abstract

Due to its multi-material capabilities, 3D inkjet printing allows for the fabrication of components with functional elements which may significantly reduce the production steps. The potential to print electronics requires jettable polymer-ceramic composites for thermal management. In this study, a respective material was formulated by functionalizing submicron alumina particles by 3-(trimethoxysilyl)propylmethacrylate (MPS) and suspending them in a mixture of the oligourethane Genomer 4247 with two acrylate functionalities and a volatile solvent. Ink jetting tests were performed, as well as thermal conductance and mechanical property measurements. The material met the strict requirements of the printing technology, showing viscosities of around 16 mPa·s as a liquid. After solidification, it exhibited a ceramic content of 50 vol%, with a thermal conductance of 1 W/(m·K). The resulting values reflect the physical possibilities within the frame of the allowed tolerances set by the production method.

## 1. Introduction

In printing several materials simultaneously, 3D inkjet printing or material jetting is currently one of the few additive manufacturing methods available, with an accuracy up to 30 µm [1,2,3]. Several vendors distribute respective commercial devices and materials [4,5,6]. They allow the printing of multicolored 3D objects for the purpose of rapid prototyping [7]. The variety in mechanical properties of the UV curable formulations ranges from flexible to comparatively stiff upon polymerization [5]. Their properties resemble, in part, typical industrial materials, like polypropylene (PP) or acrylonitrile butadiene styrene (ABS) [8,9]. A range of biocompatible polymers extend the applications into the area of dentistry [10]. The formidable structural characteristics lead to a steady transition towards the rapid manufacturing of functional components. This trend also fosters the development of new materials, which are necessary in providing properties, like thermal resistance, magnetization and electrical conductivity [11,12,13]. In the foreseeable future it might be possible to produce 3D inkjet-printed electronic devices with only a limited amount of additional assembly steps [14,15,16]. However, heat management is likely to remain an important task in ensuring a sufficiently long service life for printed devices [17,18,19]. In conventionally manufactured electronics, thermally conductive but electrically insulating ceramic materials or polymer ceramic composites are state of the art in terms of excess heat dissipation [20,21,22]. Similarly, the addition of particulate ceramics to UV or thermally curable monomer and oligomer precursors could generate suitable, thermally conductive materials for 3D inkjet printing [23,24,25].

The formulation of such composites, however, is subject to the constraints of the technology. Due to the micrometer dimensions of the printhead nozzles, the particulate additive size must be adjusted in accordance with the respective orifice. Thereby, the composite should have a surface tension in the range of 25 mN/m to 50 mN/m and a viscosity between 10 mPa·s and 40 mPa·s [3,26]. A common approach for predicting a composite’s aptitude for inkjet printing is the utilization of three dimensionless numbers [26,27,28]. These are the Weber number (*We*), Reynolds number (*Re*) and Ohnesorge number (*Oh*):

(1) $$We=\frac{\upsilon^{2}\rho a\;}{\gamma }$$

(2) $$Re=\frac{\upsilon \rho a\;}{\eta }$$

(3) $$Oh=\frac{1}{Z}=\frac{\sqrt{We\;}}{Re}=\frac{\eta \;}{{\left(\gamma \rho a\right)}^{1/2}}$$

with *ρ*, *η* and *γ* being the density, dynamic viscosity and surface tension of the fluid, respectively. The velocity of the droplet after ejection from the nozzle is denoted as v and a is the diameter of the nozzle. The parameter *Z* defines the integrity of the droplet [27]. With 1 < *Z* < 10, the parameter indicates that, when below its minimum value, the droplet is too viscose to separate from the material bulk and, when above its maximum value, it is not coherent enough and forms satellite droplets. A We number of 4 is the value at which a droplet has the minimum velocity to be ejected from the nozzle [28]. However, when the velocity is too high, splashing of the droplet occurs upon impact on the substrate, which can be predicted by the following:

(4) $$We^{1/2}Re^{1/4}=f\left(R\right)$$

with *f(R)* being a function of surface roughness, which yields a value of roughly 50 for flat, smooth surfaces. For these theoretical predictions to be valid, the material is required to be homogeneous, not showing signs of sedimentation during printing and Newtonian in nature [27].

Contrary to this, ceramic-filled composites often show a non-Newtonian behavior and an increasing viscosity with solid content, due to particle interactions [26,29]. Thermal conductivity relies upon a high solid loading. It is governed by the underlying physics of the thermal energy transport, which rises with ceramic content and gains traction after exceeding a threshold limit of 30 vol% [30,31]. A widely used theoretical approach for describing this process in highly filled composites is the Bruggeman model [22,32,33,34]:

(5) $$1-\varphi =\frac{\lambda_{F}-\lambda_{C}\;}{\lambda_{F}-\lambda_{M}}\cdot {\left(\frac{\lambda_{M}\;}{\lambda_{C}}\right)}^{\frac{1}{3}}$$

with *φ* being the ceramic content of the composite, *λ_F_* being the thermal conductance of the fillers, *λ_M_* being the thermal conductance of the matrix and *λ_C_* being the thermal conductance of the composite. Phonons, which are lattice vibrations of the materials, transfer the thermal energy over a percolated pathway and contribute significantly to *λ_C_* [35]. However, this contribution can be mitigated when the size of the particulate fillers decreases and approaches the Kapitza radius [36]. The radius defines the particle size where no improvements in the thermal conductance relative to the organic matrix can be measured due to phonon scattering at the particle matrix interface. Larger particles exhibit less interfacial area. Yet, they are prone to a higher sedimentation rate due to a decreased influence of Brownian motion in comparison to colloids, while still being subjected to gravitational forces [37]. In practice, this necessitates redispersion of the composite before use after it has sedimented [26]. The sediments are often hard and resist redispersion due to the strong interactions of the by now agglomerated fillers. A reduction of their surface energy can ease the homogenization process prior to the utilization and further decrease the sedimentation velocity in the first place [38]. Such a reduction of the ceramic surface energy can be achieved by covering it with organic moieties. The options in that direction are vast and encompass completely closed organic shells, molecular brushes, adsorbed polymers and simple coatings with small molecules [39,40,41,42]. However, an extensive surface modification also occupies physical space within the composite, so that the benefit of a decreased particle interaction is opposed by the lower content of the thermally conductive ceramic phase [43,44]. Thereby, the maximum packing density of a particulate system is roughly 65 vol% for spherical monomodal objects [45,46]. Furthermore, organic moieties can increase the thermal resistance of the ceramic interface by decreasing the tendency of the particles to percolate [47].

To satisfy the requirements on the technology and, on the material side, elaborate printhead, concepts have been developed recently using piezo-pneumatic mechanics exhibiting large nozzles of up to 100 µm with a significantly higher viscosity tolerance [16,48]. Still, the drawback of these concepts might be the reduced printing accuracy. 

To remain within the established inkjet printing technology while still being able to print composites with high solid contents, volatile solvents can be utilized. This is a practice which is the standard for the 3D inkjet printing of ceramics [49,50,51]. Thus, to avoid clogging, a rule of thumb is often employed where the particles are chosen to be roughly one 10th of the nozzle diameter being in the submicron range [26]. Brownian motion extends their duration of levitation and works against gravitational forces [37]. Widely used silane crosslinking agents can prevent agglomeration [52]. The small molecules attach after an initial hydrolysis in the presence of water molecules via condensation onto hydroxyl-rich surfaces [53]. Often they form thin layers on the ceramic, without compromising the thermal conductance [47]. Efficient functionalization takes place, among others, on Al_2_O_3_. The ceramic is available in abundance, and is therefore economically viable, and exhibits a comparatively high bulk thermal conductance of 30 W/(m·K) [54]. Besides volatile solvents, acrylate terminated, highly viscous oligourethanes are often employed as polymerizable components [55]. They exhibit decent particle-wetting properties due to their polar segments. At the same time, their polymerization is less prone to the formation of heterogeneous networks than acrylate monomers, which are often used in inkjet inks [56,57]. The energetic situation of the liquid composites is described by the DLVO theory, which is extended, among others, by investigations on polar forces [58]. This theoretical background encourages the utilization of solvents with a high polarity, which introduce charges and extensive solvent molecule attachments onto the already-functionalized ceramic surface [59]. The respective solvents often show high boiling points in combination with low vapor pressures, which prevent evaporation and clogging at the printhead nozzle [26]. After evaporation, the material exhibits an increase in the ceramic content. While it is beneficial for thermal conductance, the mechanical properties may suffer due to defects brought about by ceramic integration [60]. One of the weak points is the interphase with the organic material, which may foster crack formation when subjected to stress [61]. 

The aim of this study is the formulation of a polymer-ceramic composite, termed ceramic ink, with a high ceramic content of at least 50 vol% upon drying and solidification. Furthermore, 3D inkjet printing is moving towards rapid manufacturing structural integrity in terms of the material gains importance and this will also be a subject of investigation in this work. As opposed to our earlier publications, which primarily dealt with the improvement of mechanical properties in polymer-ceramic composites with low filler content, the goal in this work is the maintenance of the mechanical properties while increasing filler content and thermal conductance [62,63]. Furthermore, more emphasis is placed on the enhancement of the ceramic stability in the organic matrix using small molecules. Hence, the study starts with the functionalization of alumina submicron particles with silane and examines a suitable concentration of linker molecules for the prevention of particle interaction and agglomeration in the ink. In the next step, the influence of four volatile solvents with different polarities on the ceramic ink is analyzed. The solvent properties affect the viscosity, surface tension and stability of the 3D inkjet process. Once a solvent is chosen, the ratio between the alumina fillers and the polymerizable organic component is varied to adjust the ceramic content in the final composite after solvent evaporation. Finally, further characterizations of the 3D inkjet printing properties for the most suitable ink formulation are conducted.

## 2. Materials and Methods

### 2.1. Materials

Table 1 and Table 2 show the materials used in this work. The specific surface area (SSA) of the commercially particles was acquired using the Gemini VII 2390 (Micromeritics, Norcross, GA, USA), which utilizes the Brunauer–Emmett–Teller (BET) method. The investigated powders were vacuum treated before a nitrogen atmosphere was established. Based on the adsorption/desorption isotherms, the calculation of the SSA was conducted. All materials were used as received, without further purification. 

### 2.2. Ceramic Functionalization

The submicron Al_2_O_3_ particles CT3000SG (CT3000) were mechanically treated and, at the same time, surface-functionalized with 3-(trimethoxysilyl)propylmethacrylate (MPS) in order to establish a homogeneous particle size distribution (PSD). The procedure was conducted using a PM 400 planetary ball (PBM) (Retsch GmbH, Haan, Germany), with two 125 mL steel beakers cladded with YZrO_2_. The grinding balls had a diameter of 2 mm and were of the same material as the inner cladding. As depicted in Table 3, the preparation involved the five samples Sil-0 to Sil-CT3000. Each was prepared by suspending different amounts of MPS with 50 g of CT3000, 11.5 g of water, 11.5 g of ethanol and 220 g of grinding balls. in each of the beakers. The grinding time was 8 h at 200 rpm. To ease the retrieval from the beakers, another 20 g of ethanol were added to each finished sample and ball milling was resumed for further 5 min at 200 rpm. The separation of the homogenized dispersion from the grinding beakers was done using a sieve. To remove the solvents, a rotary evaporator was used, whereby 70 mL of sample was dried at a time. The bath temperature was set to 50 °C and the pressure to 200 mbar. After 100 min the pressure was changed to 30 mbar and drying continued for 20 min to remove the remaining moisture. Consequently, each dried sample was treated with a mortar and pestle.

The MPS binding efficiency was determined via thermogravimetric analysis (TGA) measurements (STA400, Netzsch GmbH, Bad Berneck im Fichtelgebirge, Germany). Around 200 mg of each of the functionalized samples was heated to 1200 °C with a heating and cooling rate of 10 (K/min) and held at the highest temperature for 30 min. The minima of the resulting curves were utilized for the calculation using the following equation:

(6) $$MPS_{coating}\left[\frac{\mathrm{mg}}{m^{2}}\right]=\frac{MPS_{hyd}\;\left[\mathrm{mg}\right]}{CT3000\;\left[m²\right]}$$

with *MPS_hyd_* being the hydrolyzed silane during the attachment to the particle surface, which is divided by the surface area of CT3000. For further analysis, the particle size distribution (PSD) was investigated via static light scattering (SLS) (LS230, Beckman Coulter, Brea, California, USA). Thereby, the powders were prepared by dispersing 0.1 g of sample in 5 g of 2-propanol, and ultrasonication was applied for 15 min. Immediately thereafter, each dispersion was pipetted into the device and analyzed. Transmission electron microscopy (TEM) images and energy-dispersive X-ray spectroscopy (EDX) of the sample Sil-CT3000 were made using a Talos F200i S/TEM (FEI Company, Hillsboro, OR, USA). The samples were inserted into the device, each being attached to a carbon coated copper grid with 400 mesh (Plano GmbH, Wetzlar, Germany), which was previously dipped directly into the respective powder, whereby the excess material was removed from the grid by shaking. Consequently, the functionalized powders were used to prepare inks by suspending them in a solution of diethylene glycol monoethyl ether (DEGMEE), Genomer 4247, diphenyl(2,4,6-trimethylbenzoyl)phosphine oxide (TPO) and dilauroyl peroxide (DLP). The compositions of these dispersions are shown in Table A1 in the Appendix A. Their preparation was conducted using a hand-held high-power stirrer (Ultra Turrax T10, IKA-Werke, Staufen im Breisgau, Germany) at 14,450 rpm for 5 min and an ultrasonic bath (Sonorex Super RK103H, Bandelin electronic GmbH and Co. KG, Berlin, Germany) for 15 min with a power of 560 W. The formulation was followed by a rheological characterization with a Bohlin CVO dynamic cone plate rheometer (Malvern Instruments, Malvern, Great Britain). The cone diameter was 60 mm, with an angle of inclination of 2°. The device was deployed at 32 °C, with a shear rate between 2 s^−1^ and 500 s^−1^. Following the measurement, half of each of the remaining inks was filtered with a 5 µm PTFE filter (Carl Roth GmbH+Co.KG, Karlsruhe, Germany) and the other half was left, as prepared, to investigate the ceramic loss during filtration using the TGA. During the analysis, 20 mg of each suspension was heated with a rate of 10 (K/min) to 900 °C, held for 15 min and cooled to room temperature with a cooling rate of 10 (K/min).

### 2.3. Ink Solvent

In this work, inks with varying volatile solvents were prepared and investigated. The composition of the inks is shown in Table A1. Sil-TEC170 was used as the MPS functionalized particles. Their preparation was conducted by applying the same parameters shown in the subchapter 2.2. The only difference was the utilization of the fillers TEC170 instead of CT3000. Both fillers were regarded as being very similar, so that the results gained during the investigation of one particle kind were transferred to the other. The volatile solvents were comprised of hexyl acetate (HexylAc), propylene glycol monomethyl ether acetate (PGMMEA), dipropylene glycol monomethyl ether (DPGMME) and DEGMEE. The preparation of the inks M-I to M-IV was completed in the same manner as described for M-0 to M-6. The samples M-I and M-II were then left for 12 h and were closed in vials so that the additionally added MPS could attach to the surface of the already functionalized particles. Consequently, all of the samples were filtered with a 5 µm PTFE filter and their rheology was recorded with the same parameters as those introduced in the previous subchapter. Surface tension measurements were done via image analysis at a pendant drop (Krüss DSA 100, KRÜSS GmbH, Hamburg, Germany). 

The materials introduced in Table A1 were subjected to jetting tests using the laboratory inkjet printer DMP 2830 (Fujifilm Dimatix Inc., Lebanon, NH, USA) with 10 pL DMC cartridges and a printhead temperature of 32 °C. During the tests, no automated cleaning cycle was utilized for the printhead. Instead, in case of nozzle occlusion, 2 s of purging and 2 µs of jetting, with subsequent retrieval and manual cleaning with a lint-free cloth and ethanol, were conducted. The cartridges were reused after each printing test by rinsing the tank and the printhead repeatedly with ethanol until no visible stains of ceramic ink were left. Additionally, the printhead was ultrasonicated in ethanol by completely immersing the piece into the solvent. Both the tank and the printhead were permanently stored in a UM400 oven (Memmert GmbH+Co.KG, Büchenbach, Germany) to dry at 60 °C.

**Figure A1:**
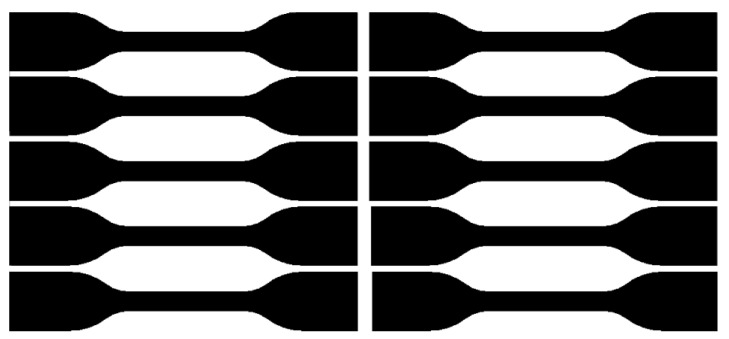
Test image for the investigation of the drop weight before and after a printing time of 18 min.

For M-I to M-IV, the ink droplet morphology was observed using the integrated Drop Watcher. To analyze the jetting stability of the sample, the drop weight was measured before and after the printing of four layers of a test image, shown in Figure A1, over a duration of 18 min. Ten nozzles were selected to jet 250,000 drops into a pre-weighted pan in order to assess the average mass. 

The wetting of M-I to M-IV was analyzed by depositing a 5-layer thick 10 mm^2^ square test image with a chosen drop spacing of 20 µm onto a PDMS substrate. In addition, in the cases of M-II, M-III and M-IV, 10 layers of the same test image were printed with a drop spacing of 40 µm onto a PDMS substrate, whereby each of the layers was dried in an oven at 200 °C for 2 min. Subsequently, the surface energies of the bare PDMS substrate and the substrate coated with the 10 layers of ink were investigated via contact angle measurement on a sessile drop of water using the Krüss DSA 100. The drop shape was fitted and the contact angles calculated via the tangential method. The same procedure was repeated with diiodomethane. The surface energies were determined using the Fowkes method. To visualize the difference in the wetting behavior between the two substrates, contact angle measurements with the ink M-IV were conducted in the same manner as was done with the previous two liquids.

Throughout the study, PDMS was utilized as substrate to enable the retrieval of the printed composites, as they show a strong tendency to adhere to surfaces.

The ceramic content of the inks can decline during ink preparation and jetting due to interactions with other surfaces. Therefore, TGA measurements were performed before and after filtration and after printing to investigate this tendency. For each of the samples and for each of the three process steps, three 20 mg subsamples were measured with a heating rate of 10 (K/min), a target temperature of 900 °C, a hold time of 15 min, and a cooling rate of 10 (K/min).

### 2.4. Ink Ratio

The ratio between the ceramic fillers and the oligomer was varied in the solvent-based inks in order to manipulate the ceramic content in the final composite after evaporation of the solvent. Samples were prepared by combining the components Sil-CT3000, DEGMEE, Genomer 4247, TPO and DLP, according to Table A3, and homogenizing them in the same manner as described for the previous subchapter for the samples M-1 to M-6 and M-I to M-IV. Similarly, each ink was filtered through a 5 µm PTFE filter and their dynamic viscosity was recorded. The surface tension was measured with the Krüss DSA100 in pendant drop conformation.

**Figure A2:**
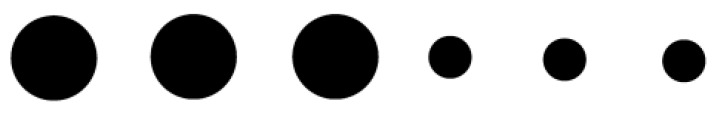
Bitmap template for the inkjet printing of specimens being used in thermal conductance measurement.

For the measurement of the thermal conductance, respective specimens were manufactured. On one hand, the four samples M-V to M-VIII have been 3D inkjet printed using the bitmap (BMT) template depicted in Figure A2, with three specimens having a diameter of 10 mm for the assessment of the thermal diffusivity and three specimens having a diameter of 5 mm for the measurement of the thermal capacitance. As substrate, a 5 mm thick aluminum sheet coated with a 100 µm thick PDMS layer was used. The layer was applied using the hand-held high precision film applicator ZUA 2000 (Proceq SA, Schwerzenbach, Switzerland) and was cured for 30 min at 60 °C. The samples were jetted by initially depositing 10 layers of ink with a drop spacing of 40 µm, with solvent drying between each layer at 200 °C for 2 min. After that, printing was done with a drop spacing of 20 µm and drying after each second layer, until 185 layers were deposited and a height of roughly 1 mm was reached. Afterwards, the specimens were placed into the Memmert oven at 100 °C for 12 h. On the other hand, four samples of casted composites have been prepared having the same shape and height using a PDMS covered aluminum substrate. The compositions for the initially liquid materials are described in Table A4. 

The samples C-10 to C-50 were produced by casting 100 µm thick arbitrarily shaped layers over each other with the hand-held high-precision film applicator until a total height of roughly 1 mm was achieved. The 2-propanol from each layer was evaporated by positioning the substrates with the samples onto a hot plate adjusted to 60 °C for 20 min. The 1 mm high samples were then cured in the Memmert oven at 100 °C for 12 h. The shape shown in Figure A2 was achieved by grinding using sandpaper.

For the assessment of the thermal conductance, three material-related values are needed: density, thermal capacitance and thermal diffusivity. The density of the samples was investigated and calculated via the Archimedes method, using Equation (7).

(7) $$Density\;\left[\frac{g}{{\mathrm{cm}}^{3}}\right]=\frac{Mass_{air}\;\left[g\right]\cdot Density_{2-Prop.}\;\left[\frac{g}{cm³}\right]\;}{Mass_{air}\;\left[g\right]-Mass_{2-Prop.}\;\left[g\right]}$$

with *Mass_air_* and *Mass_2-Prop_* being the sample mass in air and in 2-propanol, respectively, and *Density_2-Prop._* being the density of 2-propanol. The thermal capacity was measured using differential scanning calorimetry (DSC) (DSC 204 C, Netzsch-Gerätebau GmbH, Selb, Germany). Three specimens per sample have been measured. The thermal diffusivity was assessed via laser flash analysis (LFA) (LFA 427, Netzsch-Gerätebau GmbH, Selb, Germany). In total, two specimens per sample have been measured and each measurement was repeated seven times. 

**Figure A3:**
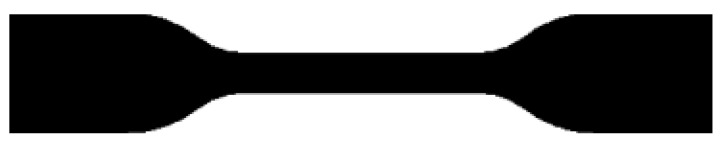
BMT template for the 3D inkjet printing of tensile specimens following DIN EN ISO 527-2 type A5 norm.

The mechanical properties of the samples have been investigated using the Z010 universal testing machine (Zwick/Roell GmbH & Co. KG, Ulm, Germany) in tensile mode. The characterization resembled the DIN EN ISO 527-2 type A5 norm. Five tensile specimens of each of the materials M-V to M-VIII were 3D inkjet printed similarly to the thermal specimens. Thereby, 150 layers were deposited until an average sample height of 1.5 mm was reached. One specimen was printed at a time, using the BMT template shown in Figure A3. The height and width of the specimens in the gauge region were measured three times with a caliper gauge. During measurement, tensile tension was applied to the specimens with an elongation rate of 1 (mm/min) until failure. The recording of the occurring forces was done with a 10 kN load cell.

### 2.5. Ink Characterization

**Figure A4:**
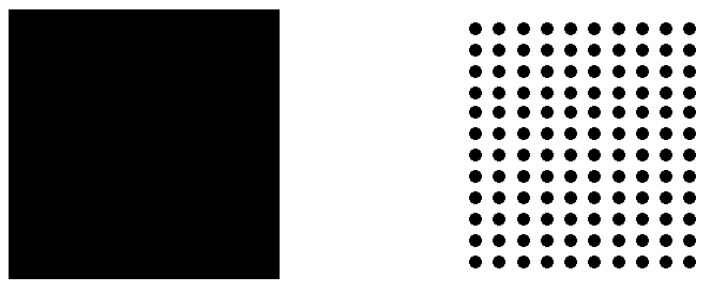
BMT template for the inkjet printing of a demonstrator with a square base and cylindrical columns on top.

The 3D inkjet printing properties for ink M-V were examined further with regard to jetting, idle time and printing temperature. Finally, a 3D test object was printed. The development of the drop morphology after ejection from the printhead nozzle was observed in the Drop Watcher of the Dimatix inkjet printer. The stroboscope camera was set to picture mode and the time of image recording was changed from 0 µs to 140 µs in 20 µs steps. The drop weight of the ink was investigated in terms of dependency of the idle time at 0, 1, 5, 10 and 20 min, by using the printer-based function of drop weight assessment. Similarly, the average drop weight was measured in terms of dependency of the printhead temperature, which was set to 32 °C, 40 °C, 50 °C and 60 °C. Finally, a demonstrator was printed with a 10 mm^2^ base and 1 mm height, with columns on top of it of the same height. The respective BMT images are shown in Figure A4. The printing was conducted in line with the previous descriptions, where initial printing was done with a drop spacing of 40 µm and subsequent volumetric printing was proceeded with a drop spacing of 20 µm.

## 3. Results and Discussion

### 3.1. Ceramic Functionalization

**Figure 1:**
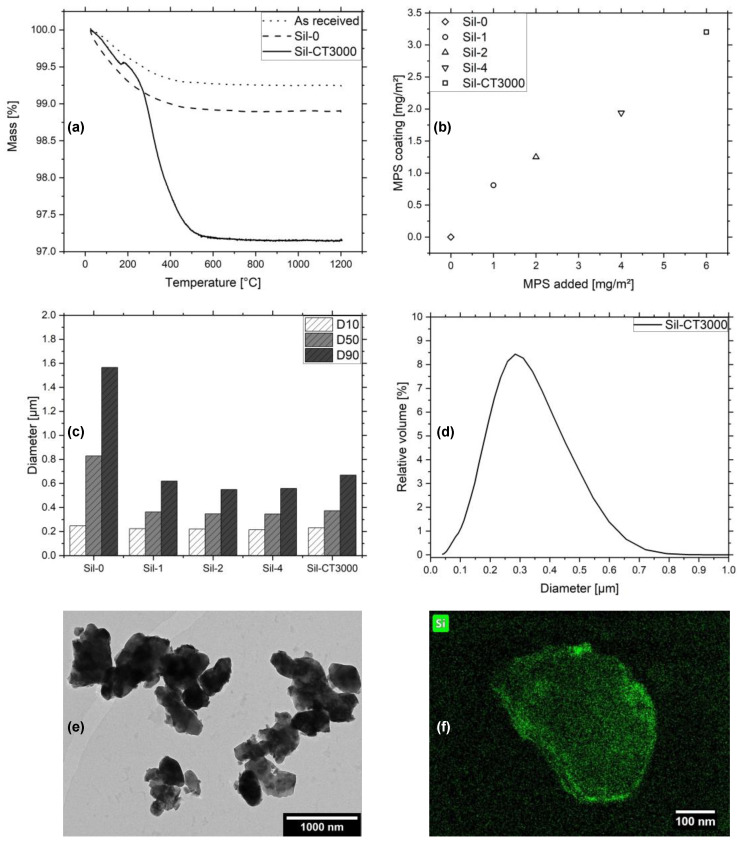
(**a**) TGA curves of CT3000 as received, Sil-0 and Sil-CT3000. (**b**) Total amount of MPS in the coating in dependency of the added MPS for Sil-0, Sil-1, Sil-2, Sil-4 and Sil-CT3000. (**c**) Particle diameters of Sil-0, Sil-1, Sil-2, Sil-4 and Sil-CT3000 expressed in D10, D50 and D90 values. (**d**) PSD of Sil-CT3000. (**e**) TEM image of Sil-CT3000. (**f**) TEM image of a Sil-CT3000 particle with an overlaid EDX signal of silicon.

The inkjet printing of particulate ceramic suspensions with a filler content of 20 vol% requires a coating of the particles to prevent them from agglomeration and clogging the nozzles. This was done by functionalization of their surfaces with the linker MPS while grinding them in a PBM. Different amounts of the linker were added to find a suitable concentration. Desorption of weakly bound molecules, probably occurring during particle drying, necessitated the monitoring of the final MPS amount using the TGA. Figure 1a shows the weight loss of the particles as received, grinded without MPS (Sil-0) and grinded with MPS (Sil-6). The as-received samples experienced a weight loss of 0.8 wt%, which is caused by the removal of surface-bound water. The same is true for Sil-0, where the weight loss of 1.1 wt% is more pronounced due to the attrition-caused increase of the surface area and surface-bound water. The H_2_O attachment first takes place by chemisorption of hydroxyl groups and then physisorption of water molecules. The OH-groups serve as anchoring points for the MPS. With 2.9 wt%, the sample Sil-CT3000 has the highest weight reduction due to the highest amount of MPS added. The results gained from the TGA analysis were used to calculate the amount of MPS per square meter, as can be seen in Figure 1b. The graph demonstrates the relationship between the MPS on the particles to the initially-added amount of the silane. It shows that the addition of 1 mg/m^2^ to 6 mg/m^2^ of the molecules resulted in 0.8 mg/m^2^ to 3.3 mg/m^2^ of attached MPS. The remaining silane is presumably composed of covalently bonded molecules and physically attached oligomolecules. Figure 1c illustrates the results of the SLS measurement of the samples Sil-0 to Sil-CT3000. The diameters are demonstrated as D10, D50 and D90 values, and visualize the filler stability improvement of Sil-1 relative to Sil-0. Sil-2, Sil-4 and Sil-CT3000, however, they do not exhibit further improvement relative to Sil-1, despite more highly utilized MPS. The highly diluted state during measurement might suffice for the particles to remain maximally suspended already, with small amounts of stabilization. The PSD of Sil-CT3000 can be seen in Figure 1d. The material is monomodal, with the peak being around 0.31 µm and the distribution ranging from about 0.05 µm to 0.99 µm. In addition, Sil-CT3000 was investigated via TEM in order to visualize the MPS coating. Figure 1e shows the irregular morphology of the sample, which, as was seen in the PSD, differs in size. Since the MPS shell is not visible, an EDX signal was projected onto a close-up image of a single particle (Figure 1f). This revealed an increased silicon concentration at the surface of the particle relative to its surroundings, which suggests that a thin MPS coating is present. 

**Figure 2:**
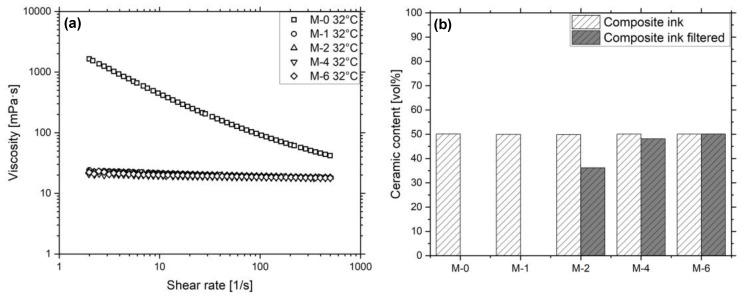
(**a**) Viscosity in dependency of the shear rate of M-0, M-1, M-2, M-4, M-6 at a temperature of 32 °C. (**b**) Ceramic content of the materials before and after filtration.

Rheological measurements of Sil-0 to Sil-CT3000 in the suspensions M-0 to M-6, as shown in Figure 2a, complement the investigation on ceramic interactions. M-0 exhibits a shear thinning behavior with a viscosity drop from 1641.6 mPa·s to 41.8 mPa·s. As already observed in the PSD measurement results, the introduction of MPS onto the particles leads to a marked decrease in particle interaction, accompanied by a reduction of the shear dependency and viscosity compared to M-0. This can be explained by the weakening of the van der Waals forces between the particles. At low shear rates the viscosities for M-1 to M-6 are between 24.2 mPa·s and 20.6 mPa·s. At a shear rate of 500 s^−1^, the values range from 18.4 mPa·s to 17.8 mPa·s. In addition to the interactions between the particles, printhead nozzle occlusion may occur due to particle interactions with the printhead material itself. Filtering tests with 5 µm PTFE filters may predict whether inks are suitable for 3D inkjet printing or not. It was shown that M-0 and M-1 cannot pass through the filter membrane. In addition, M-2 exhibited ceramic retention in the filter, leading to its occlusion during the procedure. The samples M-4 and M-6 were filterable without significant reduction of the ceramic content. Figure 2b summarizes the ceramic concentration measurement before and after the filtration of M-0 to M-6. It indicates that a sufficient MPS amount is necessary to counteract van der Waals forces and that the filler Sil-CT3000 in M-6 is the most suitable for further investigations.

### 3.2. Ink Solvent

**Figure 3:**
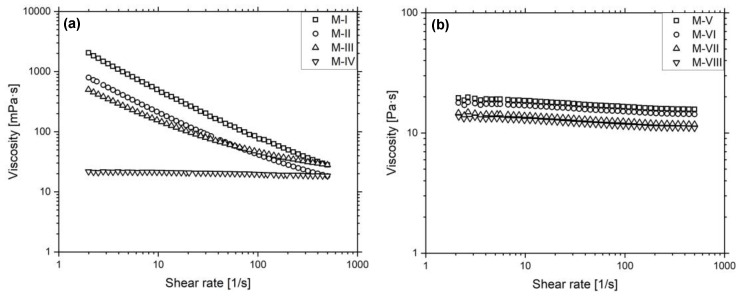
Viscosity of the filtered inks (**a**) M-I to M-IV and (**b**) M-V to M-VIII after filtration in dependency of the shear rate at a temperature of 32 °C.

The influence of volatile solvents was investigated for the inks M-I to M-IV. Figure 3a shows the rheology of the materials. The samples M-I to M-III exhibit shear thinning, while only a minor shear rate dependent viscosity reduction is visible for M-IV. The reason for that is the increasing permittivity of the solvents from HexylAc over PGMMEA and DPGMME to DEGMEE, which leads to the reduction of particle interaction. This is particularly visible at smaller shear forces, where the viscosity difference of the inks is larger. The additional amount of MPS in M-I and M-II for surface energy reduction is a direct consequence of the particle interaction, which would otherwise prohibit the filtering of the inks. Among others, Table 4 shows the surface tensions and viscosities of the inks M-I to M-IV, the utilized solvents and the oligomer Genomer 4247. The surface tension and viscosity of Genomer 4247 is the highest of all the materials, due to its high molecular weight and its high number of polar segments. The increase in polarity is also responsible for the rise of the respective values for the solvents and the inks M-I to M-IV.

At the higher temperature, all of the materials exhibit a noticeable reduction in viscosity, except for M-I and M-II, which could be explained by the crosslinking processes of the added unreacted MPS molecules. With respect to surface tension and viscosity, the measurements show that the inks, with exception of M-I, meet the requirements for inkjet printing. 

**Figure 4:**
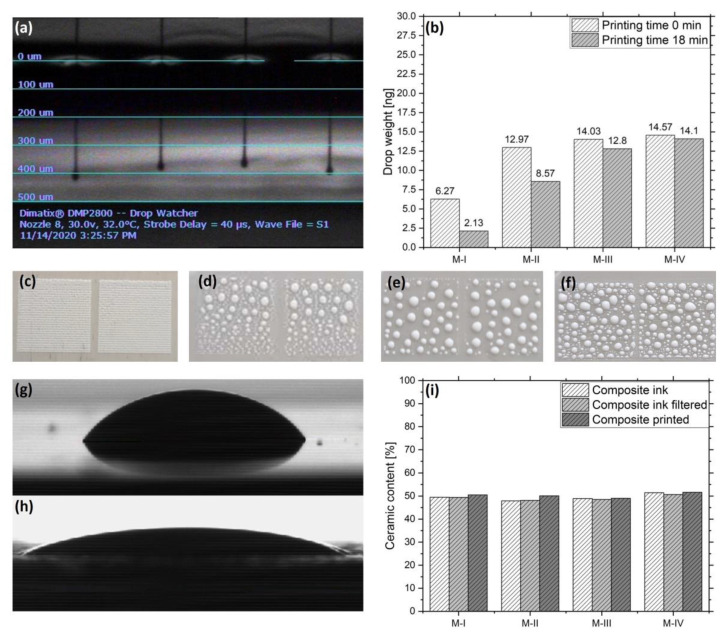
(**a**) M-IV ink during jetting in the Drop Watcher. (**b**) Measurement of the average drop weight of M-I to M-IV with 10 nozzles at 32 °C before and after the printing of a test image. (**c**–**f**) The four materials deposited onto PDMS with a drop spacing of 20 µm. (**g**) Drops of the material M-IV on PDMS and (**h**) on a previously printed and dried film of the same material. (**i**) Ceramic content of the composite ink before and after filtration, as well as after printing.

Figure 4a shows the formation of droplets in the Drop Watcher of the inkjet printer. This image is representative for the initial jetting of the inks M-I to M-IV, where no occlusion of the nozzles is visible. The drop weight of the materials after a printing time of 0 min and 18 min is illustrated in Figure 4b. The drop weight for M-I is initially 6.3 ng and decreases to 2.1 ng. Similarly, the drop weight for M-II decreases from 13.0 ng to 8.6 ng. This reduction is probably caused by the evaporation of the solvents due to vapor pressure, so that only frequent cleaning cycles can keep the materials in an operational state during printing. 

On the contrary, M-III and M-IV do not indicate significant weight changes, which means that the nozzles stay functional for a longer time. Figure 4c–f demonstrates the deposition of the four materials with a drop spacing of 20 µm onto a PDMS coated aluminum substrate. For M-I, the image indicates good wetting with a homogeneous covering of the substrate, which is caused by their comparatively low surface tensions of 16.1 mN/m relative to the surface energy of PDMS, which is 21.0 mJ/m^2^. The deposition of M-II, M-III and M-IV leads to large drop formations due to their high surface tensions of 27.9 mN/m, 30.1 mN/m and 31.7 mN/m, respectively. To improve the wetting, 10 layers were printed with a drop spacing of 40 µm to form a composite film with a surface energy of 34.4 mJ/m^2^, which allowed for significantly better surface coverage. The difference is illustrated in Figure 4g,h, where, at first, a drop of M-IV is deposited on PDMS, having a contact angle of 57.4°, and the same drop is then placed onto the composite film, which leads to a lower contact angle of 29.1°. Figure 4i displays the ceramic content of the materials before and after filtering, as well as after printing. It shows that during the processing of the materials no relevant amounts of ceramic were lost, which demonstrates the good stabilization of the particles inside the inks. 

With regular cleaning cycles, all inks have shown to be suitable for composite deposition. However, due to economic reasons, the reduction of cleaning cycles is likely to be important for volumetric printing. In this regard, the solvents HexylAc and PGMMEA are less suitable for the printing of concentrated ceramic suspensions due to their inferior stabilizing properties when compared to the other two solvents and due to their seemingly high vapor pressure. Both factors lead to a fast occlusion of the nozzles. DPGMME and DEGMEE combine good stabilizing characteristics with a subjectively low vapor pressure. The latter solvent, however, offers the best results overall, and is therefore chosen for further experiments.

### 3.3. Ink Ratio

**Figure 5:**
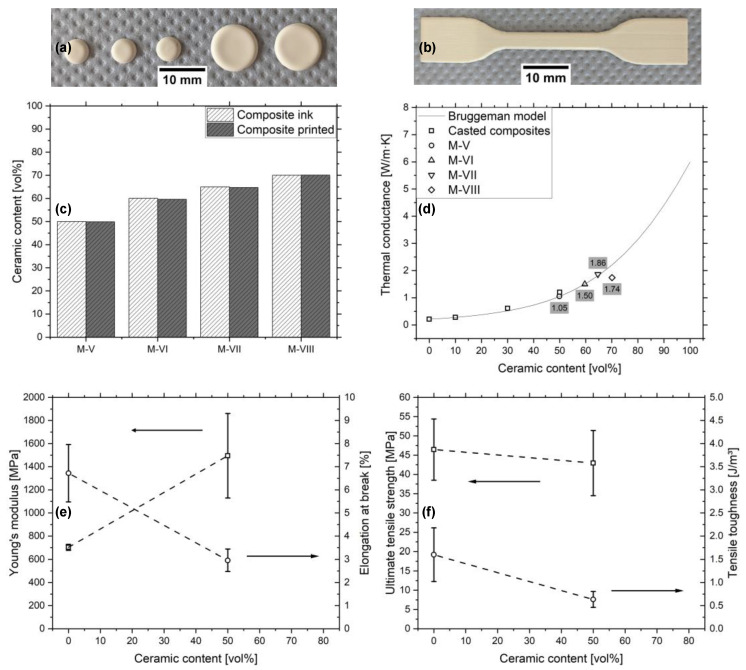
(**a**) Specimens for the thermal conductance measurement and (**b**) for the tensile test. (**c**) Ceramic content of the M-V to M-VIII inks and printed composites. (**d**) Thermal conductance of the casted composites and of M-V to M-VIII in dependency of the ceramic content complemented by the Bruggeman model. (**e**) Tensile modulus and elongation at break in dependency of the ceramic content. (**f**) Ultimate tensile strength and tensile toughness in dependency of the ceramic content.

The adjustment of the ceramic to the polymerizable organic ratio in the ink aims at increasing the ceramic content and thermal conductance in the final composite. Figure 3b shows the viscosity of the inks M-V to M-VIII in dependency of the shear rate. Just like M-IV, the materials exhibit negligible shear thinning. The influence of Genomer 4247 is noticeable and declines from sample M-V to M-VIII, while the viscosity, at 500 s^−1^, drops from 15.7 mPa·s to 11.0 mPa·s. Table 4 indicates that the viscosities decrease at the elevated temperature of 60 °C. The surface tensions of the materials stay approximately the same, with values ranging between 31.1 mN/m and 33.0 mN/m, which, therefore, require the printing of composite layers with 40 µm drop spacing for better wetting prior to the volumetric printing. Figure 5a,b show the investigated specimens produced for the assessment of the thermal conductance and the mechanical properties, respectively. Figure 5c illustrates the ceramic content of M-V to M-VIII as composite inks prior to filtering and as printed composites. The ceramic content does not decrease relative to the organic components after filtering and printing, which indicates the stability of the fillers in the inks. As intended, the ceramic content increases from 50 vol% to 70 vol%. The thermal conductance of the materials in dependency of the ceramic content is displayed in Figure 5d. The values for the casted composites C-0 to C-50 and the inks M-V to M-VIII are in the range of 0.21 W/(m·K), to 1.86 W/(m·K) and follow the Bruggeman model. Only M-VIII deviates from the model, which can be explained by the inclusion of thermally insulating air due to the exceeding of the maximally dense packing of the ceramic. Consequently, the ceramic content in the composite is possibly lower than the measured 70 vol% shown in Figure 5c, as the method does not account for air, which is a source of error. The Bruggeman model was adjusted for a filler thermal conductivity of 6 W/(m·K). The low value might result from a large proportion of small particles in the PSD, which fall below the Kapitza radius and increase the interfacial thermal resistance of the composite. Figure 5e shows the tensile modulus and the elongation at break for the printed Genomer 4247 and M-V. The stiffness increases from 702.3 MPa to 1495.0 MPa and the elongation at break (ε_max_) decrease from 6.7% to 3.0% due to the low elasticity of the ceramic fillers. Figure 5f shows the ultimate tensile strength (UTS) and tensile toughness (U_T_) of the materials. The UTS decreases from 46.5 MPa to 43.0 MPa and the U_T_ declines, influenced by the UTS and ε_max_, from 1.6 J/m^3^ to 0.6 J/m^3^. The mechanical properties of the materials M-VI to M-VIII could not be assessed, since the tensile specimens broke while being prepared. Cracks started to appear in the gauge section during the crosslinking in the oven at 100 °C. On one hand, this hints at the fact that the mechanical properties further decline with the increase of the Sil-CT3000 content. On the other hand, this shows that the shrinkage of the Genomer 4247 might be too large for highly filled composites.

The modification of thermal conductance by increasing the ceramic content in the composite is possible up to a filling grade of 65 vol%. The increase beyond 50 vol%, however, comes at the expense of the mechanical properties. Therefore, to ensure the integrity of printed components during manufacturing and handling, the ink M-V was chosen for further investigation.

### 3.4. Ink Characterization

**Figure 6:**
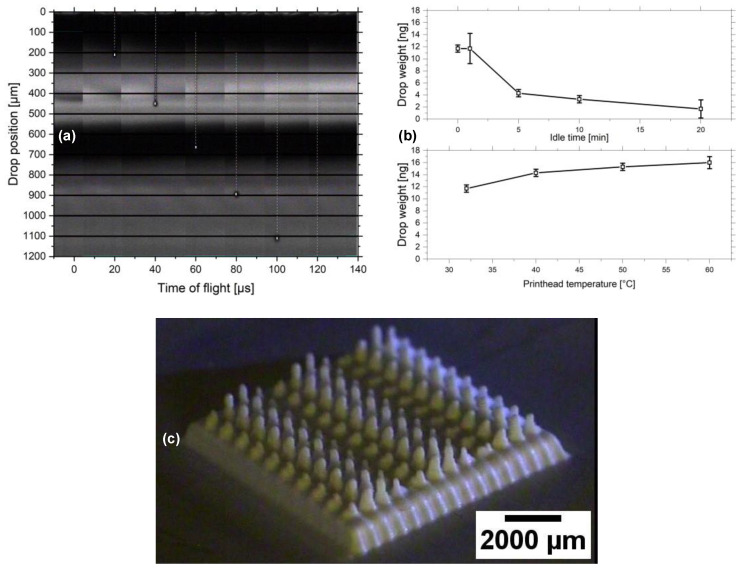
(**a**) Drop procession during jetting observed over a timeframe of 120 µs. (**b**) Drop weight of the ink measured as a function of the idle time at 32 °C and as a function of the printhead temperature. (**c**) Printed cooling element for the purpose of demonstration.

The ink M-V was further characterized regarding its 3D inkjet properties. Therefore, the nature of the ink drop flight and morphology was investigated. The drop position is shown as function of time during the ejection from the nozzle in Figure 6a. The drop velocity is 11 m/s, which, according to theory, is beneficial and will not result in splashing upon impact of the drop onto the substrate. The Weber, Reynolds and Ohnesorge numbers support this finding. Their values are 159.2, 20.8 and 0.6, respectively. In the idle state of the printhead, however, an increasing occlusion of the nozzles is observable with time. It noticeably sets in after 5 min, as Figure 6b exhibits. A cleaning cycle repeated in short consecutive intervals may prevent this negative effect. Heating of the nozzles beyond 32 °C, which also serves as risk for increasing evaporation and occlusion, does not lead to a reduction of the ejected material. Instead, more material passes through the nozzles at temperatures of up to 60 °C, as can be seen in Figure 6b, the explanation of which could be the reduction of ink viscosity. This finding was utilized to improve the jetting stability during the 3D inkjet printing of a demonstrator, which is shown in Figure 6c. The structure is a rectangular platform with an area of 10 mm^2^ and a total height of roughly 2 mm. The columns were intended to be cylindrical with a radius of 0.5 mm. Yet, the result indicates that the printing process was not precise enough to achieve the structures. Among others, a possible explanation is the misalignment during the retrieval and reinstallment of the demonstrator for solvent evaporation. In addition, the resulting column height is heterogeneous, which hints at a possible partial occlusion of some of the nozzles while the printer was idle, which were not recanalized during consecutive printing steps. 

## 4. Conclusions

In this study, a polymer-ceramic composite for 3D inkjet printing has been formulated, which exhibits a ceramic content of 50 vol% after solidification and a thermal conductance of roughly 1.0 W/(m·K). The mechanical properties show a change relative to the unfilled matrix material. The stiffness increases by 210% and the ε_max_ and UTS decrease by 54% and 7.5%, respectively, leading to a decline of the material tensile toughness by 60%. The reduction of the mechanical properties with filler content prohibits, in combination with the polymerization shrinkage, a higher ceramic concentration, which would otherwise be beneficial for the thermal conductance. Apart from that, the ink meets the stringent requirements of the printing technology and shows a high stability during jetting. Printing at elevated temperatures, as well as the short-term periods where the printhead is in its idle state, do not lead to nozzle occlusion if appropriate cleaning cycles are introduced. Therefore, the characteristics of the material should allow, in theory, an accurate print result. However, probable inaccuracies during the alignment and possible nozzle occlusions during the 3D inkjet printing of the demonstrator showed that further adjustments of the jetting process itself are necessary. 

## Figures and Tables

**Table 1:** Organic materials, which were utilized in this work, were not further purified and were used as received.

Chemical Name	CAS. No.	Supplier
3-(trimethoxysilyl)propylmethacrylate	2530-85-0	Merck KGaA
Ethanol	64-17-5	Carl Roth Gmbh
2-propanol	67-63-0	Carl Roth Gmbh
Genomer 4247	-	Rahn AG
Diphenyl(2,4,6-trimethylbenzoyl)phosphine oxide	75980-60-8	TCI CO., LTD.
Dilauroyl peroxide	105-74-8	Merck KGaA
Hexyl acetate	142-92-7	Merck KGaA
Propylene glycol monomethyl ether acetate	108-65-6	Merck KGaA
Dipropylene glycol monomethyl ether	34590-94-8	Merck KGaA
Diethylene glycol monoethyl ether	111-90-0	Carl Roth Gmbh
Diiodomethane	75-11-6	Merck KGaA

**Table 2:** Commercial particles utilized in this work.

Product Name	Chemical Name	SSA (m^2^/g)	Supplier
CT3000SG	Al_2_O_3_	6.42	Almatis GmbH
TEC170 ^1^	Al_2_O_3_	8.71	Tecnan

^1^ Material has no product name. The name is an internal designation.

**Table 3:** Samples of CT3000 which were homogenized in the PBM while being functionalized with MPS. The varying amount of the silane is shown in absolute values and relative to the total surface area of the particles.

Sample Name	MPS (g)	MPS (mg/m^2^)
Sil-0	0	0
Sil-1	0.32	1
Sil-2	0.64	2
Sil-4	1.28	4
Sil-CT3000	1.94	6

**Table 4:** Surface tension and viscosity of the materials M-I to M-VIII as well as the values for the used volatile solvents and the oligomer Genomer 4247. The viscosities were measured at 32 °C and 60 °C at a shear rate of 500 s^−1^.

	Surface Tension [mN/m]	Viscosity at 32 °C [mPa·s]	Viscosity at 60 °C [mPa·s]
Genomer 4247	38.4 ± 1.7	4223.0 ± 145.1	3231.0 ± 66.4
HexylAc	24.6 ± 0.4	1.4 ± 0.1	1.1 ± 0.0
PGMMEA	28.1 ± 1.3	1.7 ± 0.0	1.2 ± 0.1
DPGMME	30.3 ± 0.1	3.6 ± 0.0	2.0 ± 0.0
DEGMEE	34.8 ± 1.3	4.2 ± 0.1	2.3 ± 0.1
M-I	16.1 ± 0.8	27.9 ± 2.1	24.2 ± 2.4
M-II	27.9 ± 1.9	18.3 ± 1.8	18.4 ± 0.7
M-III	30.1 ± 0.9	28.1 ± 0.6	18.8 ± 1.2
M-IV	31.7 ± 0.2	18.4 ± 0.1	9.0 ± 0.1
M-V	31.1 ± 0.2	15.7 ± 0.1	7.7 ± 0.0
M-VI	32.3 ± 0.5	14.3 ± 0.1	7.0 ± 0.1
M-VII	33.0 ± 0.1	11.8 ± 0.1	6.4 ± 0.1
M-VIII	32.3 ± 0.2	11.0 ± 0.0	5.9 ± 0.0

## Appendix A

**Table A1:** Composition of solvent-based inks each with a ceramic content of 20 vol%. The purpose of the samples is the assessment of the silane coating influence.

Composition	M-0 [wt%]	M-1 [wt%]	M-2 [wt%]	M-4 [wt%]	M-6 [wt%]
Sil-0	49.2	0	0	0	0
Sil-1	0	49.5	0	0	0
Sil-2	0	0	49.6	0	0
Sil-4	0	0	0	49.9	0
Sil-CT3000	0	0	0	0	50.5
DEGMEE	37.0	37.1	37.1	37.1	37.1
Genomer 4247	13.2	12.9	12.8	12.6	12.0
TPO	0.40	0.39	0.38	0.38	0.36
DLP	0.13	0.13	0.13	0.13	0.12

**Table A2:** Composition of solvent-based inks for the investigation of the solvent influence on the material properties.

Composition	M-I [wt% (vol%)]	M-II [wt% (vol%)]	M-III [wt% (vol%)]	M-IV [wt% (vol%)]
Sil-TEC170	51.0 (20.3)	51 (21.7)	51.6 (21.9)	51.6 (22.4)
MPS	1.2 (1.7)	1.2 (1.9)	0 (0)	0 (0)
HexylAc	36.4 (62.4)	0 (0)	0 (0)	0 (0)
PGMMEA	0 (0)	36.4 (59.8)	0 (0)	0 (0)
DPGMME	0 (0)	0 (0)	36.9 (61.5)	0 (0)
DEGMEE	0 (0)	0 (0)	0 (0)	36.9 (60.5)
Genomer 4247	11.0 (14.9)	11.0 (15.9)	11.1 (16.0)	11.1 (16.4)
TPO	0.33 (0.44)	0.33 (0.47)	0.33 (0.47)	0.33 (0.48)
DLP	0.11 (0.18)	0.11 (0.19)	0.11 (0.19)	0.11 (0.20)

**Table A3:** Composition of solvent-based materials for the investigation of the ceramic-acrylate ratio after evaporation of the solvent. The ceramic content is 20 vol% in all materials.

Composition	M-V [wt% (vol%)]	M-VI [wt% (vol%)]	M-VII [wt% (vol%)]	M-VIII [wt% (vol%)]
Sil-CT3000	50.9 (22.2)	51.1 (22.2)	51.22 (22.16)	51.3 (22.2)
DEGMEE	36.5 (59.5)	40.9 (66.3)	42.5 (68.8)	44.0 (71.1)
Genomer 4247	12.1 (17.6)	7.7 (11.1)	6.0 (8.7)	4.5 (6.5)
TPO	0.36 (0.52)	0.23 (0.33)	0.18 (0.26)	0.14 (0.19)
DLP	0.12 (0.22)	0.08 (0.14)	0.06 (0.11)	0.05 (0.08)

**Table A4:** Composition of the samples C-0, C-10, C-30, C-50 for the preparation of casted composites. The ceramic content after solvent evaporation is 0 vol%, 10.1 vol%, 30.8 vol% and 52.0 vol%, respectively.

Composition	C-0 [wt% (vol%)]	C-10 [wt% (vol%)]	C-30 [wt% (vol%)]	C-50 [wt% (vol%)]
Sil-CT3000	0 (0)	24.9 (8.4)	51.2 (25.8)	74.5 (43.4)
2-propanol	0 (0)	14.8 (23.7)	10.7 (23.3)	8.5 (23.6)
Genomer 4247	96.2 (96.0)	58.0 (65.2)	31.8 (48.8)	16.3 (31.7)
TPO	2.88 (2.83)	1.74 (1.94)	0.95 (1.45)	0.49 (0.94)
DLP	0.96 (1.16)	0.58 (0.80)	0.32 (0.60)	0.16 (0.39)
